# Teaching scripts via smartphone app facilitate resident-led teaching of medical students

**DOI:** 10.1186/s12909-021-02782-w

**Published:** 2021-06-08

**Authors:** Nicholas R. Zessis, Amanda R. Dube, Arhanti Sadanand, Jordan J. Cole, Christine M. Hrach, Yasmeen N. Daud

**Affiliations:** 1 Department of Pediatrics, Northwestern University Feinberg School of Medicine, 225 East Chicago Avenue, Box 152, Chicago, IL 60611 USA; 2 Department of Pediatrics, Washington University School of Medicine, Saint Louis, MO USA; 3 Department of Pediatrics, Emory University School of Medicine, Atlanta, GA USA; 4 Department of Neurology, Washington University School of Medicine, Saint Louis, MO USA

**Keywords:** Smartphone app, Teaching scripts, Resident-led teaching, Near-peer teacher, Medical student clerkship, Pediatrics

## Abstract

**Background:**

Previous studies have suggested that resident physicians are the most meaningful teachers during the clinical clerkships of third-year medical students (MS3s). Unfortunately, residents often feel unprepared for this crucial role. The pediatrics clerkship at our institution identified a paucity in the frequency of resident-led teaching with MS3s. Lack of confidence, suboptimal teaching space, and insufficient time were cited as the most significant barriers. To enhance resident-led teaching of MS3s, we created teaching scripts of general pediatrics topics accessible via a smartphone application (app).

**Methods:**

Prior to the implementation of the app, MS3s and pediatric residents were surveyed on clerkship teaching practices. From May 2017 through July 2018, pediatric residents working with MS3s were introduced to the app, with both groups queried on resident teaching habits afterward. We compared pre-intervention and post-intervention data of time spent teaching, teaching frequency, and a ranking of pediatric resident teaching performance compared to residents of other MS3 core clerkships.

**Results:**

44 out of 90 residents (49%) responded to a pre-intervention survey on baseline teaching habits. 49 out of 61 residents (80%) completed our post-intervention survey. Pre-intervention, 75% (33/44) of residents reported spending less than 5 min per teaching session on average. Post-intervention, 67% (33/49) reported spending more than 5 min (*p* < 0.01). 25% (11/44) of residents reported teaching at least once per day pre-intervention, versus 55% (27/49, *p* = 0.12) post-intervention. Post-intervention data demonstrated a statistically significant correlation between app use and increased frequency of teaching (*p* < 0.01). The MS3 average ranking of pediatric resident teaching increased from 2.4 to 3.4 out of 6 (*p* < 0.05) after this intervention.

**Conclusions:**

Residency programs looking to reform resident-led teaching, particularly of residents early in their training, should consider our novel approach. In addition to addressing barriers to teaching and creating a platform for near-peer teaching, it is adaptable to any specialty or learner level. Future direction includes developing objective measures for teaching performance and content proficiency to better assess our intervention as an educational curriculum, as well as further investigation of the intervention as a controlled trial.

**Supplementary Information:**

The online version contains supplementary material available at 10.1186/s12909-021-02782-w.

## Background

During their clinical clerkships, medical students consistently rank resident physicians as their most influential teachers [1–9]. Medical students also tend to rate the overall quality of their clinical clerkship favorably if they work closely with and are taught by residents, a rating which is often independent of the quality of teaching itself [1, 6, 10–13]. Increasing teaching and mentoring by residents improves clerkship satisfaction and test scores among students [11, 14–17]. Clerkships with perceived insufficient teaching and residents with negative attitudes towards teaching can hinder learning and are rated less favorably by medical students [18].

Most residents do not find it burdensome to be assigned a more rigorous teaching role [14]. In fact, residents often desire to teach more while performing their clinical duties. Residents commonly report a lack of confidence, inappropriate teaching space, and insufficient time as barriers to teaching [19–22].

Smartphones and smartphone-based applications (apps) are commonly utilized in medicine, with 100% of medical students in 2015 owning a smartphone or tablet and 92% of healthcare professionals already using smartphone apps in patient care-related activities [23]. As app content is easy to access, apps are a beneficial tool for the ever-evolving hospital environment [23–28].

At our institution, third-year medical students (MS3s) had previously given feedback that they did not receive as much resident teaching during their pediatrics clerkship compared to other clerkships. To identify barriers to resident teaching, we discussed this problem with pediatric residents and MS3s, and obtained survey data. Similar to the literature [19–22], pediatric residents at our institution described a lack of confidence, inappropriate teaching space, and insufficient time as common barriers.

We were unable to find an established resource at our institution to address these barriers and needs. From the above discussions, and our own observations, we determined that MS3s and pediatric residents shared downtime when waiting for elevators prior to and after noon conference (often a five- to ten-minute wait). Conceptualizing such a setting as suitable for teaching served as inspiration to create quick teaching scripts for residents that would be advantageous in this setting. Teaching scripts are composed of a trigger, key take-away points, and teaching strategies. The trigger, in this instance a clinical vignette, prompts the teacher to select the most important teaching points [29–33].

Our solution was to create a smartphone app to house such materials on general pediatrics topics, which, through easy accessibility, we hoped would have utility in establishing new teaching environments. We hypothesized that such a resource could increase the frequency and time spent on resident-led teaching, thereby enhancing our MS3 pediatric clerkship experience.

## Methods

### Study population, outcome metrics, and institutional review board

Our study took place at Washington University School of Medicine in Saint Louis, Missouri. Third-year medical students and pediatric residents constituted our study population.

Our primary outcomes included:

1. Frequency of resident-reported teaching of medical students as measured by survey.
2. Duration of resident-reported teaching of medical students as measured by survey.
3. Medical student perception of pediatric resident-led teaching as measured by student ranking of resident teaching quality relative to the other MS3 core clerkships.

Our study was exempt from our Institutional Review Board given the voluntary nature of the survey and app use, the anonymity of survey participants, and the lack of potential for adverse impact on students’ opportunity to learn required educational content.

### Pediatric clerkship curriculum

The required MS3 clerkships include 12 weeks of surgery, 12 weeks of internal medicine, 4 weeks of neurology, 4 weeks of psychiatry, 4 weeks of a selective, 6 weeks of obstetrics and gynecology, and 6 weeks of pediatrics. Within their pediatrics clerkship at Saint Louis Children’s Hospital, a free-standing children’s hospital within a quaternary academic center, MS3s spent 2 weeks on a combined general pediatric and subspecialty inpatient floor, 2 weeks in the newborn nursery, 1 week in the pediatric emergency room, and 1 week in outpatient clinics. Our study took place on the inpatient floors and newborn nursery. At these sites, MS3s participate in daily rounds with pediatric house staff and attending physicians, as well as weekly case management conferences and bedside teaching. They also receive several hours of learning-objective guided didactic lectures from faculty each week. Students are responsible for completing several observed admission history and physical examinations, for which they receive feedback on their clinical, differential diagnosis, and written skills by faculty. They are also assessed via their overall clinical performance, professionalism, objective structured clinical examination, and end of clerkship multiple-choice National Board of Medical Educators written subject examination.

### Pre-intervention surveys

For our initial needs assessment, we surveyed both MS3s and pediatric residents to ascertain baseline teaching characteristics and perceptions within the pediatrics clerkship. In May 2017, outgoing members of the MS3 class were asked to rank their six core clerkships based on “quality of resident teaching” from one (lowest) to six (highest), a subjective task (MS3 pre-intervention survey is available in Additional file 1). MS3s who had not yet completed their core clerkships were excluded. Simultaneously, pediatric residents were queried on their baseline teaching of MS3s (resident pre-intervention survey is available in Additional file 2, although survey questions 2 and 6 did not lead to data discussed in this manuscript).

### Teaching scripts

Next, we created teaching scripts regarding general pediatrics topics, applicable both clinically and for the end-of-clerkship written examination. To select teaching script topics, the learning objectives established by the pediatric clerkship were reviewed. All chosen topics provided key content to inform these learning objectives, several of which were also selected due to a lack of coverage in the didactic clerkship lectures. Twelve teaching scripts were created: community acquired pneumonia, asthma, respiratory distress of the newborn, sexually transmitted infections, bronchiolitis, urinary tract infections, upper airway obstruction, febrile seizure, glycogen storage disorders, lysosomal storage disorders, developmental milestones, and Kawasaki disease. Multiple pediatric residents created the teaching scripts, referencing evidence-based practice. Our content was then edited and approved by the Hospitalist Education Committee within the Division of Pediatric Hospitalist Medicine at our institution, which includes many published medical educators. There were three pediatric resident leaders for this project: one to oversee the creation of teaching scripts and annually ensure the topics were kept up to date, one to oversee the resident survey data, and one to oversee the medical student data.

Teaching scripts provided organized content that could be used without preparation prior to teaching. To appeal to different learning and teaching styles, we used three formats:

- An outline format, for “chalk-talk” style teaching sessions (see Additional file 3). This is ideal for situations in which a resident wants to use a chalk or dry-erase board to organize his or her thoughts, although the outline can be used without these written mediums. The outline constitutes about a page of bulleted notes on a single topic with 3–5 main talking points. Each main point has prompts to lead the resident’s discussion. For the assessment of respiratory distress of the newborn, for instance, one of the main talking points is “History,” for which the sub-bullet points facilitate the resident to ask the medical students about how to investigate this chief concern. A concise answer is then provided in the outline if either the medical students, or resident, need further guidance.
- PowerPoint© slides (lecture format) were created for a more in-depth discussion when time allowed (see Additional file 4). This format was utilized by residents who desired to teach with a visual aid, which could be presented either via a smartphone or a computer monitor. The content generally was the most detailed of the three formats. The main talking point appears first at the top of the slide (i.e. “Pathophysiology”), the resident would then be able to discuss this point independently without additional information, then supplement it (if deemed necessary by the resident) with our provided teaching points by progressing the presentation to reveal additional details that would then appear at the bottom of the slide.
- Question-and-answer electronic cards based on a clinical vignette, to facilitate quick interactive teaching sessions (see Additional file 5). This was the format most frequently used by residents. This format did not require a visual aid or dry-erase board like the above formats. Using the app, the resident was first provided with a question to either propose to the students or trigger discussion. A discussion can ensue, with the goal of inspiring the resident to expand on important topics at their discretion or share real life patient examples. When ready, the resident and student can scroll to the next card to see the answer and teaching point together. This format more directly facilitates creativity and autonomy on the part of the resident, as they can discuss their thoughts regarding the question prompt off-script before proceeding to see the written teaching point. All topics in this format could be completed in any location within about 5 min, though longer discussions can ensue depending on the needs of the teacher and learner.

To allow for easy resident access, we compiled and uploaded our content to a unique folder on an institutional account of a healthcare resource aggregation app. Every pediatric resident in our program receives a smartphone at the beginning of residency, to be used during clinical activities. This app was pre-installed on each resident’s smartphone. Dedicated technical support was not needed, as the app was already established at our institution.

### Post-intervention surveys

Starting in May 2017, an email was sent at the beginning of each block to residents starting a rotation in which they would be expected to work closely with MS3s. This email described the teaching resources available on the app. At the conclusion of each rotation, residents were asked to complete a survey regarding the app and their medical student teaching. This process occurred monthly for 14 months. Resident teaching frequency over a four-week rotation was subjectively self-identified by survey (see Additional file 2 for post-intervention resident data, although survey questions 5, 8, and 9 did not lead to data discussed in this manuscript), with a range including “never,” “every other week,” “weekly,” “several times a week,” “daily,” or “more than once per day.” Resident average teaching duration was also subjectively self-identified (see Additional file 2), with a range including “1–2 min,” “about 5 minutes,” “about 10 minutes,” “about 15 minutes,” “about 20 minutes or more,” or “I didn’t teach at all.”

In May 2018, the MS3 class, who had been exposed to this intervention over the last year, was asked to rank their core clerkships in terms of resident teaching ability (see the post-intervention survey in Additional file 1).

### Statistical analysis

Pre-intervention and post-intervention frequency and duration of resident-led teaching of MS3s were compared using the Mann-Whitney U Test. Spearman’s Method was used to test correlation between use of our app teaching resources with frequency of and time spent teaching MS3s. MS3 rankings of the quality of pediatric resident teaching relative to other MS3 clerkships, before and after intervention, were compared using the Kolmogorov-Smirnov comparison of distributions. All statistical analyses were performed using R v.3.5.2 for Mac (R Foundation for Statistical Computing, Vienna, Austria).

## Results

The pre-intervention survey response rates were 36% (51 responses out of the 142 third-year medical student class [after excluding 10 MS3s for not having completed every required clerkship]) and 49% (44 responses out of the 90 pediatric residents at our institution). The post-intervention survey response rates were 29% (41/142) of the MS3 class and 80% (49/61) of residents.

In our pre-intervention qualitative survey, over 90% of MS3s reported resident teaching to be an “extremely important” component of their clinical education. MS3s described that the most effective resident teachers were those who offered “quick” or “brief” teaching points and generally showed an interest in medical students and their education. Only 9% of residents reported being “satisfied” or “very satisfied” with the amount of medical student teaching they were able to perform over a four-week rotation with MS3s.

**Fig. 1:**
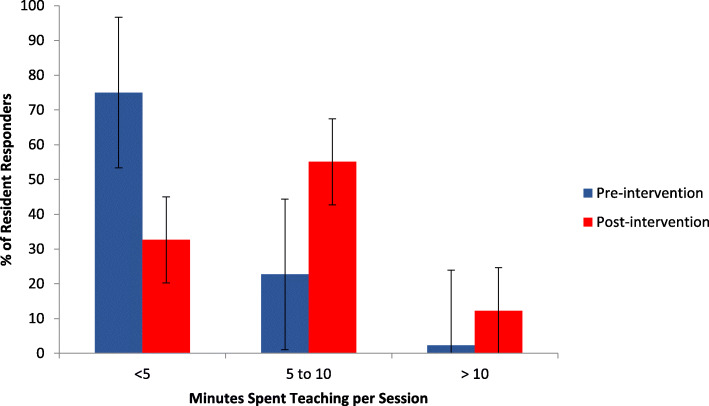
Minutes spent teaching MS3s per teaching session before (*n* = 44) and after (*n* = 49) introduction of smartphone-based teaching app. *p* < 0.01 for increased time spent teaching per session post-intervention (Mann-Whitney U Test)

**Fig. 2:**
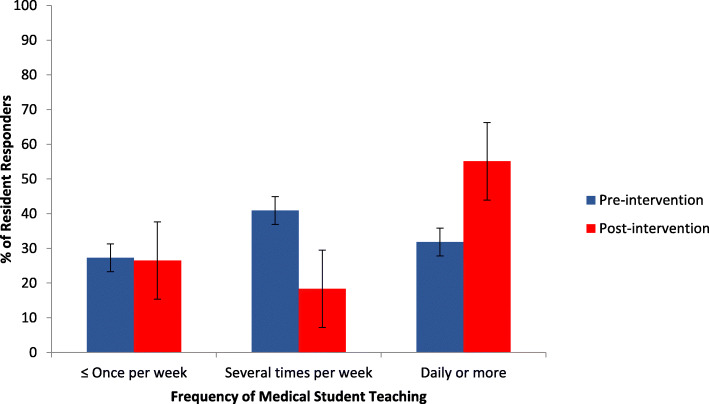
Frequency of resident-led teaching of MS3s before (*n* = 44) and after (*n* = 49) introduction of smartphone-based teaching app. *p* = 0.12 for increased teaching frequency post-intervention (Mann-Whitney U Test)

Pre-intervention, 75% (33/44) of residents reported spending less than 5 min per teaching session on average. Post-intervention, 67% (33/49) reported spending at least 5 min teaching per session (*p* < 0.01, for increased time spent teaching) (see Fig. 1). Pre-intervention, only 25% (11/44) of residents reported teaching once per day or more on average. Post-intervention, 55% (27/49) of residents reported teaching at least once per day on average, though this overall increase in teaching frequency was not statistically significant (*p* = 0.12) (see Fig. 2).

During our post-intervention analysis, 60% of residents reported using our app in at least some capacity, with 38% using it for at least half of their teaching sessions. Post-intervention data did show a significant correlation between app use and increased frequency of teaching (*p* < 0.01).

Encouragingly, 50% of residents “agreed” or “strongly agreed” that our resources better enabled them to teach medical students more reliably and more frequently. 65% of residents reported that they planned to use our app for medical student teaching in future clinical rotations.

Comparing post-graduate year one (PGY1) versus PGY3 pediatric resident cohorts, 50% of PGY1s disclosed a lack of confidence as a barrier to teaching pre-intervention, compared to just 14% of PGY3s. Additionally, 75% of PGY1s planned on using the app in the future, compared to 45% of PGY3s. Otherwise, with respect to teaching frequency and duration, as well as all other previously mentioned metrics, there were no notable differences when contrasting PGY1 and PGY3 data.

**Fig. 3:**
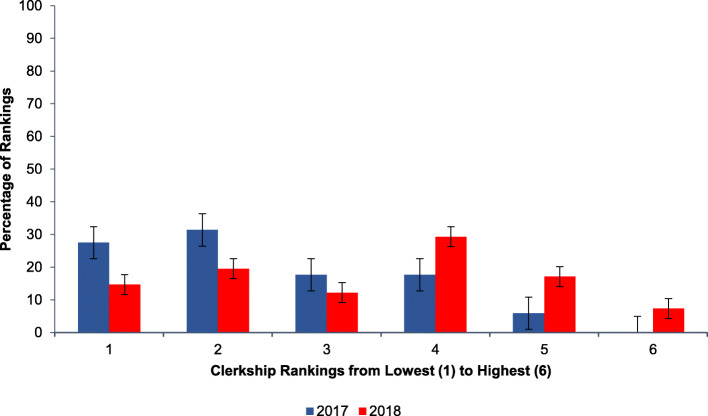
MS3 ranking of the quality of pediatric resident teaching relative to other core clerkships before (2017, *n* = 51) and after (2018, *n* = 41) introduction of smartphone-based teaching app. *p* < 0.05 for improved clerkship ratings post-intervention (Kolmogorov-Smirnov comparison of distributions)

When medical students were surveyed at the end of the first year of our intervention, they reported an improvement in pediatric resident teaching ranking compared to other clerkships. Pre-intervention, MS3s ranked pediatric resident teaching at an average of 2.43 out of the 6 core clerkships (*n* = 51). After implementation, medical student ranking of pediatric residents increased to 3.37 out of 6 (*n* = 41, *p* < 0.05 for increased clerkship ranking) (see Fig. 3).

## Discussion

### Key findings

Resident physicians are tasked with many responsibilities. In addition to caring for patients and acquiring medical knowledge for themselves, they must pass on their knowledge to medical students. Unfortunately, there are barriers to accomplishing this. To improve medical student education, our focus was to provide resources that would help residents overcome barriers to teaching, as both residents and medical students benefit from resident-led teaching. Promisingly, our results suggest several correlations with our intervention: residents engaged in longer teaching sessions, this increase in length did not come at the expense of teaching frequency, and post-intervention, medical students ranked the quality of resident teaching that they received during their pediatrics clerkship more favorably.

### Impact on resident physicians

Teaching is a crucial skill for residents to develop during their training, as it impacts their future clinical practice. Fortunately, the process is worthwhile to the teachers themselves. Near-pear teaching improves the learning of the near-peer teacher [20, 34–39]. In doing so, it prepares physicians for their future role as educators [37, 40] and may enhance their competency as clinicians [41]. Moreover, providing teaching responsibility to residents enhances leadership, communication, and organizational skills, all key elements to effective clinical practice [42–44]. The communication skills of physicians are directly linked to patient satisfaction and adherence to medical treatment [42, 44, 45], demonstrating the benefits to one’s daily clinical practice.

The concept of disseminating teaching scripts via a smartphone app offers several advantages. Smartphones are ubiquitous, easily accessible, and prior studies have shown that medical professionals, including residents, are very willing to use app technology in clinical settings [23–28]. While apps continue to be developed that offer teaching materials directly to trainees [24, 46–48], or medical information directly to clinicians [49, 50], our intervention aimed to facilitate the interaction between near-peer teachers and learners, more than providing information alone. To this end, the app did positively influence the frequency and duration of resident-led teaching. A possible advantage of our model, then, is that it may allow residents to learn teaching skills by discussing, answering questions, and engaging in a meaningful way with near-peer learners [20, 31, 34, 37].

We propose that our intervention addresses the main barriers to resident teaching during clerkships, as described by our own data and that of the literature: a lack of time, confidence, and inadequate space to teach [19–22]. By providing teaching materials via a portable medium, residents could take advantage of brief downtime in any location, which may have mitigated the time [51] and teaching space barriers [52]. In terms of self-confidence concerns, providing scripts helped fill in resident knowledge gaps and identified content at a level appropriate for MS3s [37, 53, 54]. Residents commented that they felt more confident and less anxious when using the app for teaching, factors that likely contributed to the increase in teaching, though we did not directly quantify this data. Survey responses also suggested that most residents who used the app were planning to continue to use it in future rotations with medical students.

We did not find statistically significant increases in the overall frequency of resident teaching post-intervention. A similar proportion of residents in our pre- and post-intervention group reported little to no teaching. However, residents who taught at least a moderate amount pre-intervention showed a trend toward increasing teaching frequency after our intervention, suggesting that our intervention may have been helpful for a subset, but not all, residents. The fact that PGY1 pediatric residents were more likely to describe a lack of confidence as a barrier to teaching compared to PGY3s, and that PGY1s were more likely to use the app in future rotations with medical students than PGY3s, suggests that addressing confidence is an appealing advantage of our model, particularly to trainees early in their training [22, 31].

### Impact on medical students

Optimizing resident teaching effort can significantly impact the experience of medical students. With nearly 85% of the education students receive during a clerkship coming from residents, residents are often considered the most impactful teachers [1–4, 6–9, 55]. While any fact can be found using a smartphone, our app curated information to the MS3 learner level and designed it to be delivered succinctly, which streamlines the delivery and addresses the barrier of not having sufficient time or confidence to teach.

As opposed to relying on a search engine to find information on a clinical topic, we are creating an interactive near-peer learning environment, which is one of the most effective modes of adult and medical education [31, 56–58]. Such an environment allows for socialization of medical students, provides role models [37, 59], and it enhances intrinsic motivation in students [37, 60]. Through the socialization and increased motivation of near-peer teaching, retention, application of knowledge, and academic performance all benefit compared to didactic lectures [37, 42, 61–63]. In this light, MS3s’ improved perception of our pediatrics clerkship correlated with increased resident teaching, an encouraging finding.

Interestingly, the small-group teaching interactions facilitated by our app content may be particularly useful to medical students in the wake of the severe acute respiratory syndrome coronavirus 2 (SARS-CoV-2) pandemic. The ability to use the app in an ad hoc small group setting is likely advantageous compared to curricula designed for a large classroom, and the goal of not only providing information, but more importantly, facilitating an in-person teaching interaction, is valuable in a time when much of medical education has converted to virtual and online formats [64–67].

### Limitations and future directions

Our study has several important limitations. Our data may be confounded by the fact that implementation of our resource was not the only modification to the pediatric clerkship in the 2017–2018 academic year. The most significant change was the organization of scheduled didactic lectures through the pediatric clerkship. Previously, MS3s were off the floor for didactic sessions at varying times, which confused residents as to when MS3s may be available for teaching. During this academic year, all didactic sessions were standardized to the same time on Thursday afternoons. This change may have confounded the post-intervention frequency of teaching if residents could better anticipate MS3 availability for resident-led teaching.

The Hawthorne effect may have impacted our findings [68]: it is difficult to assess the extent to which the app changed teaching habits alone versus the influence of our project email reminders and the awareness of residents that their teaching was being studied. With an observational study design, as opposed to a randomized control trial, the control group in our study becomes less robust. A pre-intervention cohort, used in this manner, is limited in its ability to reject the null hypothesis [69]. Future iterations of this study should rely on a control group that does not have access to or awareness of the app, as opposed to asking residents to describe teaching habits retrospectively (which may introduce recall bias). Doing so may minimize the influence of the Hawthorne effect [68, 70]. A control group without access to the app was not initially pursued due to logistical limitations of residents frequently rotating to other sites in the hospital, therefore making it difficult to blind the control and experimental groups from one another.

Finally, our results are limited by a small sample size from one medical school and rely on survey, precluding the inference of causality. The statistical interpretation of resident teaching ability, as ranked by clerkship from the perspective of MS3s, is limited by its nonlinear and categorical nature. For instance, one student may see a very small difference between their first and second ranked clerkship, whereas another student may have experienced a greater difference. This difference is not captured through our survey methodology. Moreover, ranking clerkships based on resident teaching quality is subjective. Future study design should provide specific qualities of resident teaching (i.e. medical knowledge, communication, etc.) for the medical students to assess [71], which may lessen the subjective nature of this survey task.

The variation in survey response, with the greatest percent response in the resident post-intervention survey, was likely due to residents receiving multiple email reminders during their month with MS3s. In all other survey groups, only a single email reminder was sent, likely contributing to low response rates. Lack of external incentives for survey completion may also have impacted response rates. Only PGY1 and PGY3 pediatric residents work with MS3s on a consistent direct basis at our institution, limiting the study population size of the post-intervention resident survey group compared to the pre-intervention cohort.

This type of intervention could be tailored for any specialty or learner level. Future endeavors include research across multiple institutions for a larger sample size and more generalizable data. Annual leadership recruitment is key to the longevity of this project, given the turnover of residency programs. Establishing resident project leaders early in their training with the responsibility of, along with oversight from faculty in the aforementioned Hospitalist Education Committee, conducting an annual review of teaching script content to ensure it is up to date. This will maintain high quality subject matter and promote longevity of the app.

Our data assessed length and frequency of teaching, but did not directly assess teaching quality or the impact of our curricular content on direct medical student learning. There is a paucity of literature on the impact of resident teaching abilities and subsequent learning achievement of medical students. Survey of both groups is helpful, but objective measures of teaching performance would be most meaningful [55]. Assessing medical student proficiency on key general pediatrics topics via a pre- and post-intervention quiz would be a beneficial future study. In doing so, the quality of resident teaching and the curriculum design, more than the quantity of teaching, could be studied.

## Conclusions

For residency programs looking to reform resident-led teaching of medical students, we propose our method as a means and framework for facilitating increased teaching effort. Such a proposal is novel in medical education, as our utilization of smartphone app technology to increase teaching of medical students by resident physicians, to our knowledge, has not been described in the literature.

We believe that to best encourage others to teach or learn, educators must create meaningful curiosity, which is a complicated task. Our proposed model uses collaboration, a worthwhile goal, and availability of resources, all to further curiosity. Information is ubiquitous in today’s age; therefore, we must create learning experiences, rather than just learning resources. In enhancing student and resident education, we are preparing them to become high-quality healthcare providers and future teachers. This is how our smartphone app derives its utility and power in the medical education arena.

## Supplementary Information

**Additional file 1.** MS3 Perception of Resident Teaching, 2017 and 2018. Medical student questionnaire.

**Additional file 2.** Pre-Intervention: Survey of Resident-Led Teaching of Medical Students, 2017; Post-Intervention: Survey of Resident-Led Teaching of Medical Students, 2017 and 2018. Pediatric resident questionnaire.

**Additional file 3.** Teaching Script Example: Respiratory Distress of the Newborn Outline Format.

**Additional file 4.** Teaching Script Example: Kawasaki Disease PowerPoint© (Lecture) Format.

**Additional file 5.** Teaching Script Example: Pediatric Community Acquired Pneumonia Question and Answer Format.

## Data Availability

The datasets used and/or analyzed during the current study are available from the corresponding author on reasonable request.

## Abbreviations

MS3: Third-year medical student
App: Application
SARS-CoV-2: Severe acute respiratory syndrome coronavirus 2
PGY: Post-graduate year
