# Supplementary material for: Teaching scripts via smartphone app facilitate resident-led teaching of medical students
Source: BMC Med Educ. 2021 Jun 8;21:331. doi: 10.1186/s12909-021-02782-w (PMC8185492; doi:10.1186/s12909-021-02782-w)
Supplement: Supplementary file 1 — Additional file 1. MS3 Perception of Resident Teaching, 2017 and 2018. Medical student questionnaire. [file 12909_2021_2782_MOESM1_ESM.pdf]

Teaching scripts via smartphone app facilitate resident-led teaching of medical students

Nicholas R. Zessis*[1], Amanda R. Dube[2], Arhanti Sadanand[3], Jordan J. Cole[4], Christine M. Hrach[2], and Yasmeen N. Daud[2]

[1]Department of Pediatrics, Northwestern University Feinberg School of Medicine, Chicago, Illinois, USA
[2]Department of Pediatrics, Washington University School of Medicine, Saint Louis, Missouri, USA
[3]Department of Pediatrics, Emory University School of Medicine, Atlanta, Georgia, USA
[4]Department of Neurology, Washington University School of Medicine, Saint Louis, Missouri, USA

*Corresponding Author:

Nicholas R. Zessis
Department of Pediatrics, Northwestern University Feinberg School of Medicine
225 East Chicago Avenue, Box 152
Chicago, Illinois, USA 60611

Telephone: 312-227-7410
Fax: 312-227-9525
Email: nzessis@northwestern.edu

Additional file 1: MS3 Perception of Resident Teaching, 2017 and 2018

MS3 Perception of Resident Teaching, 2017 and 2018

There are 5 questions in this anonymous and voluntary survey. It should take about 5 minutes to complete. Thank you for your input!

* Required

1. Have you completed all of your core clerkships (Pediatrics, Internal Medicine, Surgery, Obstetrics & Gynecology, Psychiatry, Neurology)? *

Mark only one oval.

- Yes
- No

Quality of Resident Teaching

2. Please rank the following clerkships in order of quality of RESIDENT teaching, with one being the worst and six being the best. *

Each specialty should only be ranked once, and each ranking should only be used once.

Mark only one oval per row.

	One	Two	Three	Four	Five	Six
Pediatrics						
Internal Medicine						
Surgery						
Neurology						
Psychiatry						
Obstetrics & Gynecology						

3. How much do you value resident teaching as part of your clinical education? (1 being not important at all and 5 being extremely important)

Mark only one oval.

	1	2	3	4	5	
Not important to my education at all	◯	◯	◯	◯	◯	Extremely important to my education

4. In your opinion, what qualities make a resident an effective teacher? *

5. Alternatively, what qualities make a resident an ineffective teacher? *

This content is neither created nor endorsed by Google.

Google Forms
