# Supplementary material for: Teaching scripts via smartphone app facilitate resident-led teaching of medical students
Source: BMC Med Educ. 2021 Jun 8;21:331. doi: 10.1186/s12909-021-02782-w (PMC8185492; doi:10.1186/s12909-021-02782-w)
Supplement: Supplementary file 2 — Additional file 2. Pre-Intervention: Survey of Resident-Led Teaching of Medical Students, 2017; Post-Intervention: Survey of Resident-Led Teaching of Medical Students, 2017 and 2018. Pediatric resident questionnaire. [file 12909_2021_2782_MOESM2_ESM.pdf]

Teaching scripts via smartphone app facilitate resident-led teaching of medical students

Nicholas R. Zessis*[1], Amanda R. Dube[2], Arhanti Sadanand[3], Jordan J. Cole[4], Christine M. Hrach[2], and Yasmeen N. Daud[2]

[1]Department of Pediatrics, Northwestern University Feinberg School of Medicine, Chicago, Illinois, USA
[2]Department of Pediatrics, Washington University School of Medicine, Saint Louis, Missouri, USA
[3]Department of Pediatrics, Emory University School of Medicine, Atlanta, Georgia, USA
[4]Department of Neurology, Washington University School of Medicine, Saint Louis, Missouri, USA

*Corresponding Author:

Nicholas R. Zessis
Department of Pediatrics, Northwestern University Feinberg School of Medicine
225 East Chicago Avenue, Box 152
Chicago, Illinois, USA 60611

Telephone: 312-227-7410
Fax: 312-227-9525
Email: nzessis@northwestern.edu

Additional file 2: Pre-Intervention: Survey of Resident-Led Teaching of Medical Students, 2017; Post-Intervention: Survey of Resident-Led Teaching of Medical Students, 2017 and 2018

Pre-Intervention: Questions 2 and 6 did not lead to data that was directly discussed in the manuscript

Survey of Resident-Led Teaching of Medical Students, 2017

There are 9 questions in this anonymous and voluntary survey. It should take about 5 minutes to complete. Thank you for your input!

* Required

1. What year are you? *

Mark only one oval.

- ( ) PGY-1
- ( ) PGY-2
- ( ) PGY-3

2. When you were a medical student, to what extent did you find resident-led teaching to be helpful? *

Mark only one oval.

	1	2	3	4	5	
It was not helpful at all.	( )	( )	( )	( )	( )	It was the most helpful part of the clerkship.

3. How often do you feel residents should be involved in teaching medical students while on the floors? *

Mark only one oval.

- Never
- At least once per month
- At least weekly
- Several times a week
- Daily
- More than once per day

4. In prior four-week rotations as a resident, on average, how often did you teach medical students (regardless of duration)? *

Mark only one oval.

- Never
- Monthly
- Weekly
- Several times a week
- Daily
- More than once per day
- I haven't worked with medical students as a resident yet

5. In these prior rotations, how much time did you spend on average per teaching session? *

Mark only one oval.

- 1-2 minutes
- About 5 minutes
- About 10 minutes
- About 15 minutes
- About 20 minutes or more
- I didn't teach at all

6. To what extent are you satisfied with the amount of time you were able to invest in medical student teaching? *

Mark only one oval.

- Very dissatisfied
- Dissatisfied
- Neutral
- Satisfied
- Very Satisfied

7. What are the qualities of an effective resident teacher? *

8. Alternatively, what are the qualities of an ineffective resident teacher? *

9. What are the barriers, if any, to teaching medical students at Washington University School of Medicine? *

Check all that apply.

- [ ] Medical student availability
- [ ] The medical students aren't interested
- [ ] I'm not interested in teaching
- [ ] Difficulty finding a physical location to teach
- [ ] It's not my responsibility, the fellows and attendings take ownership
- [ ] High volume of patients/not enough time
- [ ] I don't feel prepared or don't have the skills necessary to teach
- [ ] I want to teach, but I don't know what topic to teach or where to get started
- [ ] There are no barriers.

Other: [ ] ____________________

This content is neither created nor endorsed by Google.

Google Forms

<u>Post-Intervention</u>: Questions 5, 8, and 9 did not lead to data that was directly discussed in the manuscript

Survey of Resident-Led Teaching of Medical Students, 2017-2018

There are 9 questions in this anonymous and voluntary survey. It should take about 5 minutes to complete. Thank you for your input!

* Required

1. What year are you? *

   Mark only one oval.

   - PGY-1
   - PGY-2
   - PGY-3

2. Over the last 4 weeks, how frequently did you teach medical students on average? *

   Mark only one oval.

   - Never
   - Every other week
   - Weekly
   - Several times a week
   - Daily
   - More than once per day

3. How much time did you spend on these teaching sessions on average? *

Mark only one oval.

- ( ) 1-2 minutes
- ( ) About 5 minutes
- ( ) About 10 minutes
- ( ) About 15 minutes
- ( ) About 20 minutes or more
- ( ) I didn't teach at all

4. Of your teaching sessions to medical students, about what percentage of them did you utilize our resources in the Dorsata app? *

Mark only one oval.

- ( ) 0%
- ( ) 1-24%
- ( ) 25-49%
- ( ) 50-74%
- ( ) 75-100%

5. Which of our teaching script content did you use the most? *

Mark only one oval.

- ( ) Powerpoint (in traditional format)
- ( ) Powerpoints (in Q&A format)
- ( ) Notecards (in outline format)
- ( ) I didn't use any of them

6. To what extent do you agree with the following: The teaching scripts enabled me to initiate more frequent and reliable teaching of medical students. *

Mark only one oval.

- Strongly Disagree
- Disagree
- Neutral
- Agree
- Strongly Agree

7. On future rotations with medical students, do you anticipate utilizing these resources in any capacity? *

Mark only one oval.

- No
- Unsure
- Yes

8. In what ways are these tools helpful? *

9. In what ways could these tools be improved? *

This content is neither created nor endorsed by Google.

Google Forms
