# Supplementary material for: Teaching scripts via smartphone app facilitate resident-led teaching of medical students
Source: BMC Med Educ. 2021 Jun 8;21:331. doi: 10.1186/s12909-021-02782-w (PMC8185492; doi:10.1186/s12909-021-02782-w)
Supplement: Supplementary file 3 — Additional file 3. Teaching Script Example: Respiratory Distress of the Newborn Outline Format. [file 12909_2021_2782_MOESM3_ESM.pdf]

Teaching scripts via smartphone app facilitate resident-led teaching of medical students

Nicholas R. Zessis*[1], Amanda R. Dube[2], Arhanti Sadanand[3], Jordan J. Cole[4], Christine M. Hrach[2], and Yasmeen N. Daud[2]

[1]Department of Pediatrics, Northwestern University Feinberg School of Medicine, Chicago, Illinois, USA
[2]Department of Pediatrics, Washington University School of Medicine, Saint Louis, Missouri, USA
[3]Department of Pediatrics, Emory University School of Medicine, Atlanta, Georgia, USA
[4]Department of Neurology, Washington University School of Medicine, Saint Louis, Missouri, USA

*Corresponding Author:

Nicholas R. Zessis
Department of Pediatrics, Northwestern University Feinberg School of Medicine
225 East Chicago Avenue, Box 152
Chicago, Illinois, USA 60611

Telephone: 312-227-7410
Fax: 312-227-9525
Email: nzessis@northwestern.edu

Additional file 3: Teaching Script Example: Respiratory Distress of the Newborn Outline Format

This format is ideal for situations in which a resident wants to use a chalk or dry-erase board to organize his or her thoughts, although the outline can be used without these written mediums. The outline constitutes about a page of bulleted notes on a single topic with 3-5 main talking points. Each main point has prompts to lead the resident's discussion. For the assessment of respiratory distress of the newborn, for instance, one of the main talking points is "History," for which the sub-bullet points facilitate the resident to ask the medical students about how to investigate this chief concern. A concise answer is then provided in the outline if either the medical students, or resident, need further guidance.

Case – A newborn nursery nurse pages you about a 1-day old boy with retractions.

I. **Ask the medical students, "What additional HISTORY would you like?"**
    a. Gestational age? Maternal risk factors for sepsis? Maternal diabetes? Meconium noted at delivery? Prenatal ultrasound findings (oligohydramnios, structural lung abnormalities, CDH)?

II. **Ask the students, "What would you like to know about the EXAM?"**
    a. Vitals: respiratory rate (tachypnea = RR > 60 breaths per minute), oxygen saturation, heart rate, blood pressure
        i. Also note color, capillary refill
    b. Pulmonary exam: work of breathing, breath sounds (bilateral vs unilateral, diminished, extra sounds – stridor or wheeze)
        i. Increased work of breathing – nasal flaring, retractions, grunting
            1. Indicate respiratory (alveolar) etiology
        ii. Tachypnea without increased work of breathing
            1. Potential cardiac etiology
        iii. Hyperpnea (deep sighing respirations)
            1. Suggests metabolic acidosis (sepsis, shock, inborn error of metabolism)
        iv. Absent breath sounds on one side → order CXR, transilluminate to assess for PTX while awaiting CXR

III. **Ask, "What LABS/IMAGING are indicated?"**
    a. CXR – helps differentiate between parenchymal lung disease (HMD, PNA, MAS) from pleural (effusion, PTX)
    b. Blood gas – can follow pH and pCO_2
    c. CBC and differential WBC – help evaluate for sepsis
        i. Blood culture is gold standard for ruling out sepsis
            1. Ask, "What limitations exist in neonatal blood cultures?"
                a. Smaller human being = less blood sample = more difficult to rule out sepsis

IV. **Treatment – varies by etiology**
    a. Discuss how you've managed prior babies with respiratory distress
    b. A large spectrum of respiratory support from supplemental O_2 to intubation, surfactant, ECMO

V. **Abbreviated Differential Diagnosis**
    a. Respiratory
        i. TTN – early respiratory distress, term/late-preterm, impaired fetal lung fluid clearance

        1. Supportive therapy, resolution in 24-72 hours
    ii. RDS/HMD – deficiency of alveolar surfactant (risk factors: prematurity, maternal diabetes)
        1. Treatment often involves intubation and exogenous surfactant
            a. Initiate antibiotics in infants with RDS since PNA may present similarly and CXR findings may be indistinguishable
    iii. MAS – meconium causes a chemical pneumonitis and inactivates surfactant, air leak is common
        1. Treatment is supportive, often requires surfactant, can require ECMO
    iv. PNA – GBS most common, caused by transplacental spread
        1. Treatment – ampicillin and gentamicin
    v. Air leaks – PTX, pneumomediastinum, pmeumopericardium, PIE
    vi. CDH – often diagnosed prenatally, left side most common, respiratory distress from pulmonary hypoplasia and pulmonary hypertension
        1. Often requires ECMO
    vii. Cardiac
        1. Shock, duct dependent systemic circulation, pulmonary overcirculation
    viii. Other
        1. Sepsis, inborn error of metabolism, cold stress, increased ICP, urea cycle disorders

Abbreviations: CBC complete blood count; CDH congenital diaphragmatic hernia; CRP C-reactive protein; CXR chest X-ray; ECMO extracorporeal membrane oxygenation; GBS group-B streptococcus; HMD hyaline membrane disease; ICP intracranial pressure; MAS meconium aspiration syndrome; NPV negative predictive value; PNA pneumonia; PPHN persistent pulmonary hypertension of the newborn; PTX pneumothorax; RDS respiratory distress syndrome; TTN transient tachypnea of the newborn; WBC white blood cell count.

References

Reuter S, Moser C, Baack M. Respiratory distress in the newborn. *Pediatrics in Review.* Oct 2014, 35 (10) 417-429; DOI: 10.1542/pir.35-10-417

Washington Manual of Pediatrics. White AJ. Second Edition. p. 82-85

Last Updated – January 2018
