# Supplementary material for: Teaching scripts via smartphone app facilitate resident-led teaching of medical students
Source: BMC Med Educ. 2021 Jun 8;21:331. doi: 10.1186/s12909-021-02782-w (PMC8185492; doi:10.1186/s12909-021-02782-w)
Supplement: Supplementary file 4 — Additional file 4. Teaching Script Example: Kawasaki Disease PowerPoint© (Lecture) Format. [file 12909_2021_2782_MOESM4_ESM.pdf]

Teaching scripts via smartphone app facilitate resident-led teaching of medical students

Nicholas R. Zessis*[1], Amanda R. Dube[2], Arhanti Sadanand[3], Jordan J. Cole[4], Christine M. Hrach[2], and Yasmeen N. Daud[2]

[1]Department of Pediatrics, Northwestern University Feinberg School of Medicine, Chicago, Illinois, USA
[2]Department of Pediatrics, Washington University School of Medicine, Saint Louis, Missouri, USA
[3]Department of Pediatrics, Emory University School of Medicine, Atlanta, Georgia, USA
[4]Department of Neurology, Washington University School of Medicine, Saint Louis, Missouri, USA

*Corresponding Author:

Nicholas R. Zessis
Department of Pediatrics, Northwestern University Feinberg School of Medicine
225 East Chicago Avenue, Box 152
Chicago, Illinois, USA 60611

Telephone: 312-227-7410
Fax: 312-227-9525
Email: nzessis@northwestern.edu

Additional file 4: **Teaching Script Example: Kawasaki Disease PowerPoint © (Lecture) Format**

This format was utilized by residents who desired to teach with a visual aid, which could be presented either via a smartphone or a computer monitor. The content was a hybrid of the outline format (described above) and question-and-answer cards (below), but generally was the most detailed of the three formats. The main talking point appears first at the top of the slide (i.e. "Pathophysiology"), the resident would then be able to discuss this point independently without additional information, then supplement it (if deemed necessary by the resident) with our provided teaching points by progressing the presentation to reveal additional details that would then appear at the bottom of the slide.

Kawasaki Disease

1

Learning Objectives

- By the end of this presentation, third-year medical students will be able to:
  – Obtain a focused history for a child with Kawasaki Disease
  – Recognize the clinical presentation
  – Understand basic management

2

Epidemiology

- One of the most common vasculitides in childhood
- Most common in children living in east Asia or of Asian descent
- More common in boys than girls
- >90% of cases in children <5 years
- Rare in children <6 months

3

Why do we care?

- Symptoms overlap with many other conditions
- Significant morbidity and mortality if not diagnosed in a timely matter

4

Pathophysiology

- Systemic, inflammatory illness that affects medium-sized arteries, especially the coronary arteries
- Inflammation of multiple tissues, but long-term sequelae in the arteries
- Destruction of elastin and collagen fibers, loss of structural integrity of the arterial wall -> dilatation and aneurysm formation

5

Pathology

- Inflammatory cell infiltration into vascular tissues
- Destruction of luminal endothelial cells, elastic lamina, and medial smooth muscle cells in severe cases

6

Case

A 3 yo boy presents with 5 days of fever that does not improve with antipyretics

What other signs and symptoms would you look for?

7

History and Physical

Diagnosis requires the presence of fever lasting ≥5 days, combined with at least four of the five following physical findings, without an alternative explanation

- Bilateral bulbar conjunctival injection
- Oral mucous membrane changes, including injected or fissured lips, injected pharynx, or strawberry tongue
- Peripheral extremity changes, including erythema of palms or soles, edema of hands or feet (acute phase), and periungual desquamation (convalescent phase)
- Polymorphous rash
- Cervical lymphadenopathy (at least one lymph node >1.5 cm in diameter)

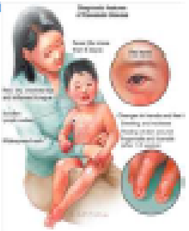

8

Initial Labs

- Complete blood counts with differential white blood cell (WBC) counts
- Aspartate transaminase (AST) and alanine transaminase (ALT)
- C-reactive protein (CRP) and erythrocyte sedimentation rate (ESR)
- Urinalysis

9

Lab Findings

- Elevation of acute-phase reactants (ESR, CRP)
- Thrombocytosis that generally develops after the seventh day of illness
- Leukocytosis, and a left-shift
- Sterile pyuria (missed on UA because leuk esterase will be negative)
- Mild to moderate increase in transaminases

10

Other Possible Lab Findings

CSF may display a mononuclear pleocytosis without hypoglycorrhachia (low CSF glucose)

- Arthrocentesis of involved joints typically demonstrates a pleocytosis
- Hyponatremia (increased risk of coronary artery aneurysms)

11

Other than Kawasaki…

- What are some possible etiologies for his symptoms?

12

Differential Diagnosis

- Viral illness
- Toxic Shock Syndrome
- Rocky Mountain Spotted Fever
- Stevens-Johnson Syndrome
- Systemic Juvenile Idiopathic Arthritis

13

Complications

- Shock
- Cardiovascular complications
  - Coronary artery aneurysms (1 in 5 patients)
  - Myocardial abnormalities
  - Infants <1 year at greatest risk
- Macrophage activation syndrome, also called secondary hemophagocytic lymphohistiocytosis [HLH]
  - May lead to severe complications including disseminated intravascular coagulopathy, cytopenias, and thrombosis

14

Initial Treatment

- IVIG within the first 10 days decreases risk of coronary aneurysms (CA) 5 fold
- Decreased CA aneurysms if given IVIG with aspirin compared to aspirin alone
- IVIG given once and patient observed to confirm resolution of fever

15

Referrals/Consults

- Pediatric rheumatologists
- Infectious disease specialists
- Cardiologists, and/or
- Hospitalists at a pediatric institution with experience in treating Kawasaki disease can aid in diagnosis and management

16

Prognosis

- Mortality due to KD is rare among children treated with intravenous IVIG
- Long-term morbidity is primarily related to the degree of coronary artery (CA) involvement
- Recurrence of KD is uncommon.

17

Follow-up

- Monitoring for recurrence of fever
- Repeat echocardiograms to assess for cardiac involvement

18

References

- Lin M.T., Wu M.H. The global epidemiology of Kawasaki disease: Review and future perspectives. *Glob Cardiol Sci Pract* 3 (2017). Doi: 10.21542/gcsp.2017.20
- Dietz, S.M., van Stijn, D., Burgner, D. *et al.* Dissecting Kawasaki disease: a state-of-the-art review. *Eur J Pediatr* 176, 995–1009 (2017). Doi: 10.1007/s00431-017-2937-5
- Newburger J.W., Takahasi M., Burns J.C. Kawasaki Disease. *J Am Coll Cardiol* 67 1738-1749 (2016).

19
