# Supplementary material for: Teaching scripts via smartphone app facilitate resident-led teaching of medical students
Source: BMC Med Educ. 2021 Jun 8;21:331. doi: 10.1186/s12909-021-02782-w (PMC8185492; doi:10.1186/s12909-021-02782-w)
Supplement: Supplementary file 5 — Additional file 5. Teaching Script Example: Pediatric Community Acquired Pneumonia Question and Answer Format. [file 12909_2021_2782_MOESM5_ESM.pdf]

Teaching scripts via smartphone app facilitate resident-led teaching of medical students

Nicholas R. Zessis*[1], Amanda R. Dube[2], Arhanti Sadanand[3], Jordan J. Cole[4], Christine M. Hrach[2], and Yasmeen N. Daud[2]

[1]Department of Pediatrics, Northwestern University Feinberg School of Medicine, Chicago, Illinois, USA
[2]Department of Pediatrics, Washington University School of Medicine, Saint Louis, Missouri, USA
[3]Department of Pediatrics, Emory University School of Medicine, Atlanta, Georgia, USA
[4]Department of Neurology, Washington University School of Medicine, Saint Louis, Missouri, USA

*Corresponding Author:

Nicholas R. Zessis
Department of Pediatrics, Northwestern University Feinberg School of Medicine
225 East Chicago Avenue, Box 152
Chicago, Illinois, USA 60611

Telephone: 312-227-7410
Fax: 312-227-9525
Email: nzessis@northwestern.edu

Additional file 5: **Teaching Script Example: Pediatric Community Acquired Pneumonia Question and Answer Format**

This format did not require a visual aid or dry-erase board like the above formats. Using the app, the resident was first provided with a question to either propose to the students or trigger discussion. Neither the resident (unless the material were reviewed ahead of time, which is not a requirement) nor the students can see the subsequent card with the answer. A discussion can ensue, with the goal of inspiring the resident to expand on important topics at their discretion or share real life patient examples. There is more creativity and autonomy in this format on the part of the resident, not being as restrained to a single script. All topics in this format could completed in any location, most of which could be completed within several minutes.

Pediatric Community Acquired Pneumonia
Q&A Format
Washington University School of Medicine
Last Updated: July 2018

1

Case

- A 6-month-old male, full term, fully vaccinated, and previously healthy, presents to the ED with 3 days of fevers to 102, new onset tachypnea, and retractions
- On exam, decreased breath sounds are heard in the LLL with crackles

2

What is your differential diagnosis?

3

Differential Diagnosis

- Community acquired pneumonia (CAP)
- Bronchiolitis
- Wheeze/Asthma
- Foreign body
- Allergic reaction
- Aspiration
- Pulmonary edema (cardiogenic vs noncardiogenic)
- (also sepsis, ingestion/irritant inhalation, non-accidental trauma)

4

What are 4 common bacterial causes of CAP in babies < 28 days old?

5

- GBS
- *E. coli*
- *Klebsiella*
- *Listeria* (less common)

6

What are common causes of CAP in infants and children < 5 years old

7

- Viruses most common (RSV, paraflu, human metapneumovirus)
- *S. pneumo*
- *S. aureus*
- *H. flu*

8

What are 3 common bacterial causes of CAP in infants and children > 5 years old?

9

- *Mycoplasma pneumonia* is the most common cause of CAP in teenagers and young adults
- *S. pneumo*
- *Chlamydia pneumonia*

10

What are common bacterial causes of aspiration pneumonia?

11

- Anaerobic oral flora
- *Peptostreptococcus*
- *Fusobacterium*
- *Bacteroides*

12

How might you distinguish bacterial vs. atypical vs. viral pneumonia based on history and exam?

(HINT: fevers, acuity of onset, associated symptoms, and auscultation findings)

13

- Bacterial:
  - Abrupt onset, often higher fevers, focal findings on auscultation, few other symptoms
- Viral:
  - Gradual onset, often preceding URI, lower fevers, diffuse/bilateral auscultation findings
- Atypical:
  - Variable onset, variable fevers, wheeze common on exam
  - Mycoplasma can cause rash, urticaria, hemolytic anemia, arthritis, pancreatitis, hepatitis, and other constitutional findings

14

What workup would you consider for suspected CAP?

15

Workup

- CAP is a clinical diagnosis, no workup is necessary
- Consider CXR for severity, inconclusive history/exam, concern for complications, or recurrence
- Consider CBC, CMP, blood culture, sputum culture for severity or to rule out sepsis

16

What are potential complications of CAP?

17

Complications

- Effusion/empyema
- Necrotizing pneumonia (often S. pneumo)
- Abscess
- Pneumatocele
- Hyponatremia (etiology unclear)

18

What outpatient antibiotics would you use…

… for < 5 years old?

… for > 5 years old?

19

Outpatient Empiric Antibiotic choices

- < 5 yo:
  - **Amoxicillin x 7days** is optimal because of great *S. pneumo* and MSSA coverage (also gets 70% of *H. flu*)
- > 5 yo:
  - Amoxicillin is still a good choice
  - Can add or substitute **azithromycin** if high concern for atypicals

20

Questions?

21

References:

- Management of Pediatric Community-acquired Bacterial Pneumonia. Amanda I. Messinger, Oren Kupfer, Amanda Hurst, Sarah Parker. *Pediatrics in Review* Sep 2017, 38 (9) 394-409; DOI: 10.1542/pir.2016-0183
- John S. Bradley, Carrie L. Byington, Samir S. Shah, Brian Alverson, Edward R. Carter, Christopher Harrison, Sheldon L. Kaplan, Sharon E. Mace, George H. McCracken, Matthew R. Moore, Shawn D. St Peter, Jana A. Stockwell, Jack T. Swanson; The Management of Community-Acquired Pneumonia in Infants and Children Older Than 3 Months of Age: Clinical Practice Guidelines by the Pediatric Infectious Diseases Society and the Infectious Diseases Society of America, *Clinical Infectious Diseases*, Volume 53, Issue 7, 1 October 2011, Pages e25–e76, https://doi.org/10.1093/cid/cir531

22
